# *Clerodendrum serratum* extract attenuates production of inflammatory mediators in ovalbumin-induced asthma in rats

**DOI:** 10.3906/kim-2107-22

**Published:** 2021-11-04

**Authors:** Poonam ARORA, Shahid Husain ANSARI, Lalit Mohan NAINWAL

**Affiliations:** 1 Department of Pharmacognosy and Phytochemistry, School of Pharmaceutical Education and Research, Jamia Hamdard, New Delhi, India; 2 Department of Pharmacognosy and Phytochemistry, SGT College of Pharmacy, SGT University, Gurugram, Haryana, India; 3 Department of Pharmaceutical Chemistry, School of Pharmaceutical Education and Research, Jamia Hamdard, New Delhi, India; 4 Department of Pharmacy, School of Medical & Allied Sciences, G. D. Goenka University, Sohna, Haryana, India

**Keywords:** *Clerodendrum serratum* extract, asthma, ovalbumin, cytokines, phytochemicals

## Abstract

In the present study, ethanolic extract of *Clerodendrum serratum* roots was investigated for its potential to reverse some features of bronchial asthma in ovalbumin-induced murine model of asthma. *Clerodendrum serratum* commonly called bharangi, (family Solanaceae) is a well-known anti-allergic drug in Asian folk system of medicines. In the present work, pharmacological studies are done to provide scientific evidence for therapeutic potential of plant in allergic asthma. Asthma was induced in experimental rats with allergen suspension of ovalbumin and aluminum hydroxide followed by treatment with dexamethasone (2.5 mg/kg, po) or *C. serratum* root extract (0.53 and 5.3 mg/kg, b. w., po). Biomarkers of inflammatory response including cell counts, immunoglobulin E, cytokines such as interleukin (IL) -4, -5, -1β, tumor necrosis factor-α (TNF-α), leukotriene (LTD-4), and nitrite concentration in blood as well as bronchial (BAL) fluid were tested. Lung functions in asthmatic and treated animals were evaluated as breathing rate and tidal volume. Treatment with *C. serratum* extract markedly (p < 0.001, p < 0.01, and p < 0.05) diminished infiltration of inflammatory cells, IgE, cytokines, and nitrites in blood serum and bronchial fluid. Improvement in lung functions (p < 0.05) of asthmatic animals after CSE treatment also supports our findings. Results of the study suggest therapeutic potential of *C. serratum* in allergic asthma that can be related to ability of plant to attenuate response of inflammatory cells and thereby, production of inflammatory and proinflammatory cytokines in airways.

## 1. Introduction

Bronchial asthma is a very common noncommunicable lung disorder characterized by chronic inflammation, excessive mucus formation, reversible constriction, and hyperresponsiveness of airways usually triggered by allergens [1]. Another pathological feature of asthma is airway remodeling, described as structural and functional changes in lungs attributed to persistent inflammatory response in airways [2–5]. Annual increase in asthma prevalence by approximately 3.6% makes the disease a global issue of public health [6]. The classical dogma of asthma is central to CD4^+^T mediated T-helper type 2 (T_H_2) cells production and IgE driven airway hyperresponsiveness. T_H_2-induced immune response is advocated to production of large array of cytokines, mainly, IL-4, IL-5, and IL-13 and manifests in clinical pathology as high IgE antibodies and eosinophil levels in asthmatic individuals [7]. Together with T_H_2 cells, innate lymphoid group 2 cells (ILC2) also play a key role in augmenting T_H_2 response in the airways. These latter immune cells express transcription factor GATA3 and govern production of type T_H_2 related cytokine, IL-5 [8].

**Figure 1:**
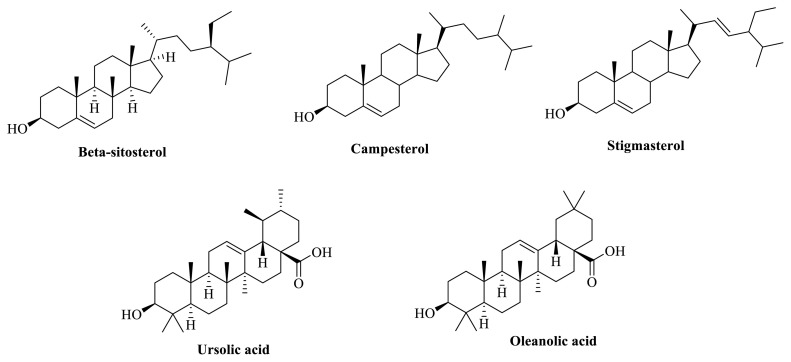
Major active phytoconstituents present in the *C. Serratum*.

Glucocorticoids, widely accepted as main stay of asthma therapy, act by modulating T_H_2 response and nonspecific antiinflammatory response, and suppress innate immune response in individuals. Effects of corticosteroids on the cellular and humoral immunity are mediated via T_H_1 cells. Treatment with other antiinflammatory drugs can provide benefit only to some extent in chronic patients. Despite several medical treatments available in the form of short-term and long-term relievers, there occurs an urgent need to seek more clinically effective targeted therapies in bronchial asthma. For decades, herbal drugs have been utilized as a source of new therapeutically active molecules, scaffolds, pharmacophores, and chemotypes. Among numerous drugs of natural origin, the genus *Clerodendrum* has been used ethnomedicinally for treatment of many diseases such as asthma, rheumatism, leucoderma in many countries of Asian continent. The biological effects of various species of *Clerodendrum* including *C. serratum* has been related to numerous category of bioactive principles including, terpenoids (serratumin A, serratoside A and B, oleanolic acid (Figure 1), queretaroic acid; flavonoids and their glycosides (7, 40-trihydroxy-30-methoxyflavone, 6,7-trihydroxy-40-methoxyflavone; 7-glucopyranoside, scutellarein, apigenin-7-O-glucuronide; steroids (a-spinasterol, stigmasterol, campesterol, β-sitosterol) and others as cinnamic acid, iscosahydropicenic acid, ursolic acid, squalene, methyl palmitate, hexadecenoic acid [9–16]. These phytocompounds have exhibited considerable pharmacological activities including antifertility, anticancer, antioxidant, hepatoprotective, immunomodulator, antiiflammatory, wound healing, and spermicidal in preclinical studies [17–24]

The plant forms an important constituent of several traditional antiasthmatic herbal formulations. However, the role of the plant in context to modulation of inflammatory markers in allergic asthma has never been investigated. In our study, we have investigated the potential of *C. serratum* standardized root extract (CSE) in ovalbumin induced airway inflammation in asthmatic animals.

## 2. Materials and methods

### 2.1. Chemicals

All the reagents of analytical grade were purchased from commercial Sigma Aldrich suppliers and were used in the research work. Standard ELISA kits of interleukins, IL-4, IL-5, IL-1β, leukotriene, LTD-4, immunoglobulin E (IgE), tumor necrosis factor TNF-α and calorimetric kit of nitric oxide (NO) were used to estimate the levels of these biomarkers in treated and control rat groups.

### 2.2. Preparation and standardization of plant root extract

*C. serratum* roots were harvested from the institutional botanical and medicinal garden of Jamia Hamdard, New Delhi, India. The harvested roots were collected, washed with water to remove unwanted debris, and dried under shade. The plant sample was validated by Dr. Singh, NISCAIR, India, with voucher number NISCAIR/RHMD/consult/-2011-12/1752/52. The dried roots (250 g) were coarsely grounded and thoroughly extracted with a mixture of ethanol and water (7: 3) for 24 h at room temperature (32 ± 2°C) with periodic stirring. The extracted content was filtered under vacuum. Filtrate was concentrated under reduced pressure to obtain semisolid crude extract. The crude extract was placed in lyophilizer to remove any residual solvent and dried at −40 °C to obtain brown colored dried powder. The lyophilized powdered extract was stowed in a desiccator until further use.

The percentage yield (%w/w) obtained of the dried extract was 37.8 ± 2.06. All the quality control parameters were performed for standardization of extract as per the guidelines of Ayurvedic Pharmacopoeia of India [12]. Standardized extract was stored at 2–8 °C till further use in experiments. For conducting pharmacological studies with CSE, 5 g of lyophilized dried powder was suspended in 100 ml of 0.1% carboxymethyl cellulose to make stable suspension for oral administration in animals.

### 2.3. Investigation of *C*. *serratum* standardized extract (CSE) in ovalbumin sensitized and challenged asthmatic rats

Thirty healthy male Wistar rats were weighed and divided randomly into five groups (6 rats per group). All the animals were shifted and allowed to acclimatize for 15 days under standard housing conditions (25 ± 2 °C/ 50 ± 5% with a 12 h light/dark cycle) before commencement of the experiment. The animals for the study were issued and approved by the Institutional Animal Ethics Committee (Approval no. JHAEC/173/CPCSEA dated 28.1.2000), Jamia Hamdard, New Delhi, India.

Group 1 (N) rats served as normal control and received vehicle (0.1% CMC w/v in purified water). Group 2 (S) rats served as asthma control; Group 3 (S + D), received standard drug, 2.5 mg/kg; Group 4 (S + CSE 1) and Group 5 (S + CSE 2) rats received CSE 0.53 mg/kg and 5.3 mg/kg, body weight (b. w.), respectively. Treatments were given daily through oral route from day 1 to 28 between 10.00 and 11.00 AM. Administered dose of the *C. serratum* root extract in rats was derived from the daily recommended dose of the drug recommended in Ayurvedic Pharmacopoeia of India [25]. Standard reference drug used in study was corticosteroid dexamethasone.

Asthma was induced in rats (group 2 to 5) by administering suspension of ovalbumin (40 mg/ animal) and Al(OH)_3_ (2 mg/ rat) [26, 27]. The suspension of ovalbumin and aluminum hydroxide acts as an allergen that activates the allergic response in the rats; and makes them sensitized and susceptible to asthma.

After 15 days of ip administration of allergen suspension, sensitized rats were exposed to mist of 1% chick egg albumin (ovalbumin) suspension (1 mg/ 100 mL normal saline) for 20 min in a closed chamber regularly (once in a day) for the next 8 consecutive days (day 15 to day 22) and thereafter, with a gap of 3 days i.e. on day 25 and day 28. In control group animals (Group I), animals which were not sensitized by allergen (ovalbumin and aluminum hydroxide), the animals were exposed to mist of normal saline solution for the above specified time period.

### 2.4. Lung function test

Lung function was gaged using two parameters (i) breathing rate and (ii) tidal volume. Rats were anaesthetized with sodium pentobarbitone (ip, 105 mg/kg) on 28th day, 5 min after challenging the OVA-sensitized rats. To record airflow signals of rat’s lung, rat’s trachea was connected with spirometer (pneumotachograph) and differential pressure transducer device [28]. The transducer integrates the electronic airflow signals into lungs tidal volume (VT, mL/s) and breathing rate (*f*, breaths/min). Physiological respiratory data was collected, analyzed with the help of Power Lab System and LabChart Programme software (spirometer Model no: FE141, ADInstruments, Pty, Australia). Before recording data in L/s, the spirometer was calibrated, and any drift in signals attributable to the instrument transducer was neutralized for high precision. Changes in *f* and VT were measured after intravenous (iv) administration of vehicle (in normal control group) and methacholine (1.5 mg/kg) in rats through femoral vein. Heparin (0.5%) was administered to prevent any chances of blood coagulation during study. One data point was generated by recording and averaging 10–12 respiratory cycles. To avoid disturbances arising due to spontaneous respiration, an iv injection of vecuronium bromide at dose of 0.2 mg/kg was given to the anesthetized rats. The excessive bronchial secretion produced during the procedure was discharged by mean of a small polyethylene tube so that the trachea was not disturbed throughout the procedure [29].

### 2.5. Collection of bronchial fluid (BAL) fluid

Bronchial fluid or bronchoalveolar fluid (BAL) fluid was collected as per procedure described by Arora et al. [29]. Briefly, lungs were lavaged with 0.9% physiological normal saline solution. The whole procedure was repeated thrice with fresh 5 mL of physiological solution. All the collected BAL fluids were mixed together and centrifuged at 4500 rpm/4 °C. Supernatant was collected to analyze inflammatory biomarkers. The cell pellet obtained after centrifugation was suspended in normal saline (1 mL) and used for quantitative analyses of total and differential leukocyte count in BAL fluid as described earlier [30].

### 2.6. Estimation of cells count in blood and BAL fluid

The blood was collected via cardiac puncture using 19-gauge sterile syringe. Two separate vials were used for blood collection. In one vial, anticoagulant (heparin) was added and stored at 4 °C to determine total blood cells and differential cell count. In the second vial, blood was allowed to clot and serum was collected through centrifugation. Serum was stored at −80 °C and was used for estimation of IgE and cytokines levels.

### 2.7. Estimation of nitric oxide, nitrites and cytokines in serum and BAL fluid

The levels of various key inflammatory biomarkers like IgE, IL-4, IL-5, IL-1β, LTD-4, and TNF-α in serum and BAL fluid were quantitatively assessed through ELISA kits. The assays were performed according to the instructions supplied with the commercial kits. Automatic ELISA reader (Model no. ELX-80MS, Biotek, USA) was used to analyze the samples. The UV kinetic method was employed to quantify the concentration of nitrite and nitric oxide in serum and BAL fluid.

### 2.8. Statistical analysis

Pharmacological responses are represented as mean ± SEM. To validate the results statistically with statistical differences at 5% level of probability (p < 0.05), one-way analysis of variance (ANOVA) followed by post hoc Tukey’s test was employed. All statistical calculations and analyses were done using Graph Pad Prism software (San Diego, CA, USA).

## 3. Results

### 3.1. Effect of CSE on lung function parameters

**Figure 2:**
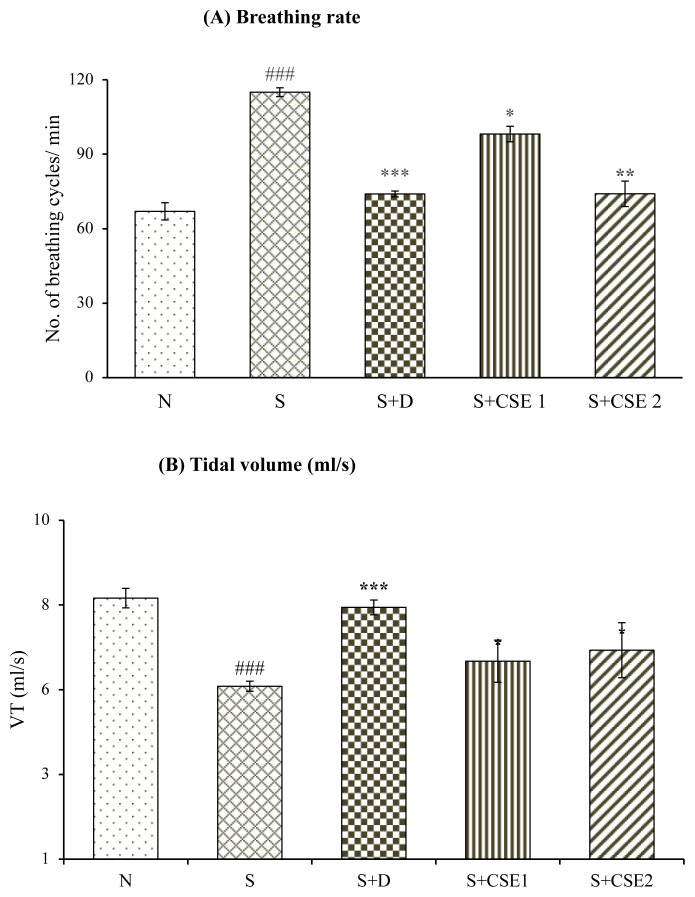
Statistical analysis of effect of CSE on lung functions in different groups. Breathing rate (A) and tidal volume (B). Values are represented as mean ± SEM. ^*^p < 0.05, ^*^p < 0.01 and ^***^p < 0.001, ns, compared to the asthmatic control (S) group; ^###^p < 0.001 compared to normal control (N) group.

Breathing rate increased substantially (p < 0.001) in asthma control animals (S) on comparing with normal control animals (N) after administration of methacholine. Treatment of animals with *C. serratum* ethanolic extract (CSE), 0.53 mg/kg and 5.3 mg/kg, b.w. elicited reduction in breathing rate by 14.65% and 35.60%, respectively, (p < 0.05) on comparison with asthma control group (S). Remarkable (p < 0.001) decrease in lungs tidal volume of asthma control group (S) after comparing with normal control group (N) was observed. Treating asthmatic rats with CSE (5.3 mg/kg, b.w.) significantly (p < 0.05) improved VT by 17.12% after comparing with asthmatic control group (Figure 2). Oral treatment of asthmatic animals with reference standard, corticosteroid, also improved lung functions significantly (p < 0.001) in comparison to asthmatic control group (S). Effect of CSE treatment in normalizing the pulmonary functions was comparable to the effects produced by reference standard drug with statistical difference between them.

### 3.2. Effect of CSE on circulating cell count in blood and BAL fluid

Various inflammatory cells including total leukocyte count (TLC) and differential cell count (DLC), lymphocytes, eosinophils, and neutrophils in blood and BAL fluid samples of asthmatic rats increased considerably (p < 0.001) in comparison to normal control (N) group). Lesser number of lymphocytes were observed in blood sample of allergen sensitized and challenged rats in comparison to normal group (N). Treatment with CSE (5.3 mg/kg, p < 0.05) and dexamethasone (p < 0.01 and p < 0.05) normalized the circulatory cells count in blood as well as BAL fluid in comparison to asthma control group (Tables 1 and 2). Reversal of OVA induced altered levels of circulatory cells after CSE treatment was found to be comparable with that of dexamethasone in both body fluids analyzed in the present study.

### 3.4. Effect of CSE on serum cytokines levels

**Figure 3:**
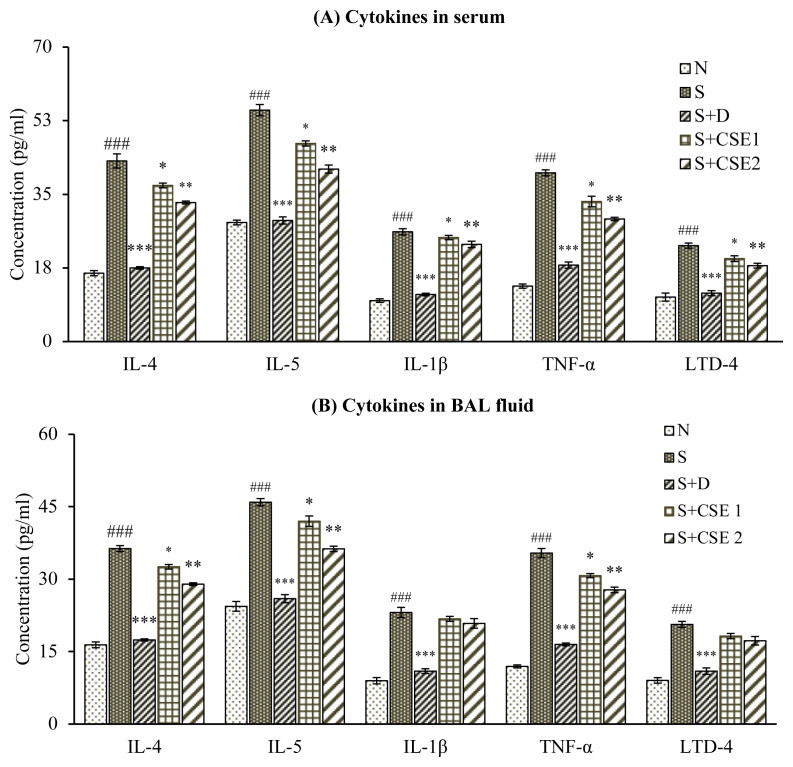
Statistical analysis of effect of CSE on various estimations in different groups; (i) serum cytokines levels (A) and BAL fluid cytokines levels (B) of different treatment groups. Data represent mean ± S.E.M. ^*^ p < 0.05, ^*^p < 0.01, and ^***^ p < 0.001, ns, compared to the asthmatic control (S) group; ^###^ p < 0.001 compared to normal control (N) group.

Sensitization and provocation with ovalbumin elicited significant (p < 0.001) increase in concentration of various cytokines, IL-4, LTD-4, IL-1β, IL-5 or TNF-α in serum of experimental asthmatic rats when compared to nonasthmatic control group (N). CSE treatment (0.53 or 5.3 mg/kg) reduced serum levels of IL-4 (18.17%, p < 0.05 and 29.21%, p < 0.01), IL-5 (21.81%, p < 0.05 or 30.6%, p < 0.01), TNF-α (14.71%, p < 0.05 or 30.32%, p < 0.01), IL-1β (8.06%, p > 0.05 or 17.11%, p < 0.05), and LTD-4 (19.18% or 18.54%, p > 0.05) in comparison to asthmatic control group (Figure 3A). Similar comparison for S+D group showed significantly reduced concentration of all the cytokines (p < 0.001) tested in our study. Comparing the efficacy of CSE with standard reference dexamethasone, we found that herbal drug was comparable in preventing OVA-induced changes in serum cytokines in rats; however, the comparison was significant at both dose levels tested in our study.

### 3.5. Effect of CSE cytokine levels in BAL fluid

Level of all inflammatory and proinflammatory cytokines in bronchial fluid of asthmatic animals were found markedly high (p < 0.001) in comparison to normal control group (N). Treatment with both doses of CSE (0.53 mg/kg and 5.3 mg/kg) elicited substantial reduction in levels of IL-4 (11.88% and 30.54%); IL-5 (15.28% and 34.08%); TNF-α (13.86% and 38.62%) and dexamethasone (p < 0.05 and p < 0.01) compared to corresponding cytokines levels in asthmatic rats (Figure 3B). CSE treatment was able to produce considerable improvement in concentration of IL-1β and LTD-4 in BAL fluid with statistical significance of p < 0.05 and p < 0.01 respectively, after comparing with asthma control group (S) animals. In comparison to standard reference dexamethasone, *C. serratum* root extract was found to be significantly comparable in reducing BAL fluid levels of cytokines in OVA-sensitized animals.

### 3.6. Effect of CSE on IgE levels of serum and BAL fluid

**Figure 4:**
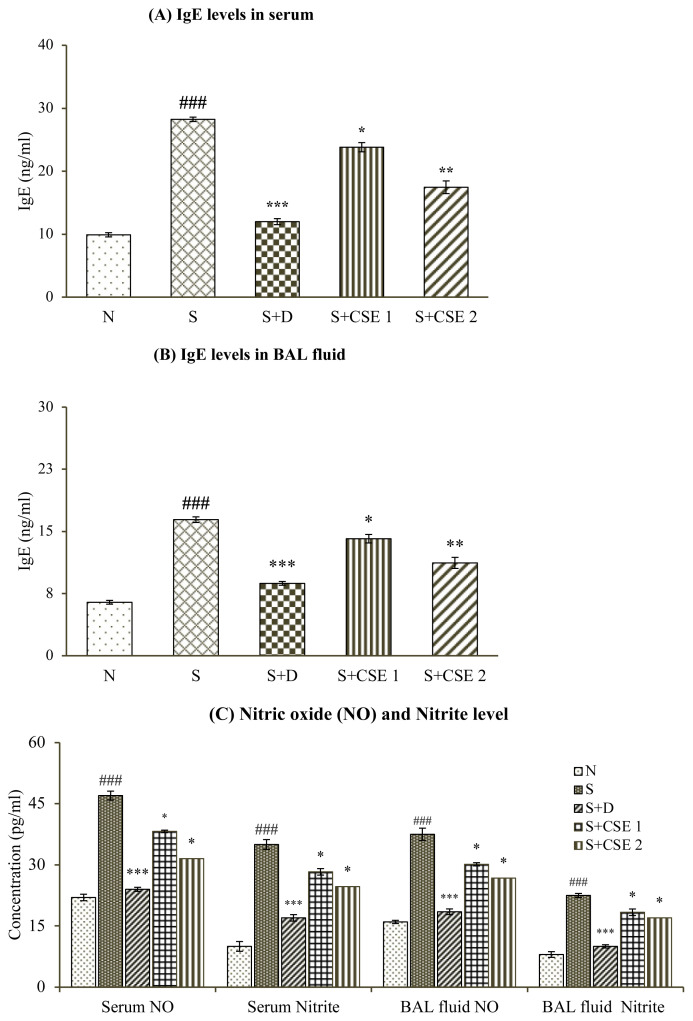
Statistical analysis of effect of CSE on various estimations in different groups (i) serum IgE levels (A); (ii) BAL fluid IgE levels (B); (iii) nitric oxide (NO) and nitrites serum and BAL fluid levels (C). Values are represented as mean ± SEM. (n = 6); ^###^ represents p < 0.001 compared to normal control (N); * represents p < 0.05, ** represents p < 0.01, and *** represents p < 0.001, compared to the asthmatic animals (S).

IgE levels of both serum and BAL fluid samples of animals increased significantly (p < 0.001) after OVA-sensitization and challenge. Significant (p < 0.05 and p < 0.01) reduction was observed by 15.68% or 38.208%, and 14.12% or 31.78% in serum and BAL fluid samples of rats receiving CSE (0.53 and 5.3 mg/kg, b.w.) after comparing with asthma control group (S). Treatment of asthmatic animals with standard drug, dexamethasone also elicited significant (p < 0.001) reduction in IgE levels in both the body fluids analyzed (Figures 4A and 4B). Furthermore, effect of herbal drug treatment in attenuating serum and BAL fluid levels of allergen-induced IgE levels was found to be statistically significant when compared with reference, dexamethasone.

### 3.7. Effect of CSE on total nitric oxide and nitrite concentration

In asthmatic rats (S), total NO and nitrite concentrations in body fluids were increased significantly (p < 0.001) in comparison to normal group (N). Animals receiving CSE at dose of 0.53 mg/kg b.w. exhibited reduction in nitric oxide in serum and BAL fluid by 18.68% and 19.62% while nitrile content was reduced by 19.17% and 18.35% in serum as well as BAL fluid (p < 0.05), respectively (Figure 4C) in comparison with asthmatic control group (S). Reference drug, dexamethasone, and higher dose of extract also showed significant reduction in elevated levels of both the analytes (p < 0.001). Reduction in serum and BAL fluid levels of total NO and nitrite after CSE treatment was comparable to standard drug treatment.

## 4. Discussion

The recent resurge in interest of researchers to utilize plants as a source of novel therapeutically active interventions can be advocated to presence of multidimensional chemical structures with varied biological functions. Due to complex nature of chronic diseases, e.g., asthma, the treatment strategy has shifted to multitarget approach” with combination agents. Spasmogenic and contractile responses of tracheobronchial smooth muscles is tightly regulated by neurotransmitters and other biological chemicals including acetylcholine, histamine, and bradykinin [31]. In sensitized individuals, allergen exposure activates naive T-cells to produce more T_H_2 cells in comparison to T_H_1. Activated T_H_2 cells by producing cytokines and other biologically active molecules are key components in the pathogenesis of asthma. Among these cytokines, IL-4 and IL-5 are mainly implicated in cardinal features of asthma, maturation of eosinophils and goblet cell, IgE mediated hypersensitive response, mucus secretion, bronchial constriction, and tissue remodeling [32].

Upon allergen recognition, and cross-linking with high affinity FcɛRI (FcepsilonRI) present on mast cells and the low affinity receptor FcɛRII or CD23 cells present on antigen presenting cells activate mast cell [33]. Activation results in the release of primary and secondary chemical substances (e.g., histamine, heparin, and proteases) and inflammatory mediators, such as cytokines and arachidonic acid metabolites [34]. Lately, it has been found that newly formed Ag-FcɛRI-IgE (allergen-FcɛRI-IgE) complex also accelerates production of chemokine ligand 28 (CCL28) that selectively attracts T_H_2 lymphocytes [35].

IL-4 has widely been reported to play central role in establishing the basis for IgE-mediated hypersensitivity reactions. IL-4 also upregulates the transcription factor, GATA-3 in naïve-T cells, thereby regulating differentiation of allergen-specific T_H_2 cells [36]. IL-5 production in airways favors the production, survival, accumulation, and activation of eosinophils in the lungs [37].

In present study, allergen sensitization elicited marked alteration in breathing rate and lung tidal volume indicating clinical symptoms of asthma. All the asthmatic control group animals (S) have shown all the symptoms and manifestations of asthma that includes coughing, irritability, and breathing difficulty. Methacholine-induced bronchoconstriction was prevented by CSE and dexamethasone treated animals, displaying broncho-relaxant effects of the drugs that can be assumed to be due to reduction in airway inflammation, thereby diminishing lung resistance to air flow.

In the study, allergen provocation of sensitized rats altered normal count of inflammatory cells, eosinophils, lymphocytes, and neutrophils in blood as well as BAL fluid of experimental animals when compared with normal control group. Oral dose of dexamethasone and CSE (5.3 mg/kg) for 28 days in asthmatic rats efficiently suppressed allergen-induced inflammation cascade and migration of immune cells particularly of eosinophils in rat’s lung, demonstrating prospective role of the herbal extract in allergen mediated eosinophilic airway disorder. Eosinophils are the cell type associated with T_H_2 immune response in asthma and have pleiotropic effects on various inflammatory cells [38]. Several pieces of studies demonstrate that controlling eosinophils in sputum of allergic patients is effective in decreasing asthma exacerbations.

T_H_2 mediators, TNF-α, IL-5, IL-4, IL-1ß play primary role in coordinating various inflammatory mechanisms in asthma. Therapeutic approaches targeting IL-4, IL-5, and TNF-α through their selective inhibitors are under clinical trials in management of asthma. In our study, presence of T_H_2 cytokines in asthmatic animals indicates perpetuating airway inflammation in sensitized lungs. Oral administration of CSE (0.53 or 5.3 mg/kg) to rats reversed IL-4, -5, and TNF-α levels in both body fluids (serum and BAL) of animals in comparison to asthma group. Effects produced by CSE at higher dose were significant and comparable to that of corticosteroid used as standard drug in study (2.5 mg/kg). However, in the study, standardized CSE extract treatment also ameliorated OVA-induced levels of IL-1β and cysteinyl leukotrienes (LTD-4) in serum and bronchioalvelolar secretion of asthmatic animals, and results were found to be statistically significant as compared to OVA-control group. Cysteinyl leukotrienes (cys LTs) represent biologically active arachidonic acid metabolites and account for features of asthma [39]. Cysteinyl leukotrienes mediate biological effects through G-protein receptors CysLTR (1–3). CysLT1R is mainly present in bronchial muscles and has a higher affinity to LTD-4. Market available montelukast, zafirlukast specifically antagonise CysLT1R only [40].

Our findings indicate that oral administration of CSE (0.53 and 5.3 mg/kg, b.w.) could neutralize the symptoms of asthma via reversing release of T_H_2 induced proinflammatory and inflammatory cytokines, the key players in pathogenesis of asthma. Here, IL-4 is directly involved inT_H_2 cell differentiation and production of downstream signals, IL-5, IL-13. Marked reversal of LTD-4 levels after treatment of asthmatic animals with CSE demonstrates possible antagonistic effects of herbal extract at CysLT1R similar to market drugs.

Serum İmmunoglobulin E (sIgE) levels are high in asthmatic individuals as compared to normal subjects and these levels increase with severity of asthma making it a strong clinically predisposing factor in the bronchial asthma. Oral administration of CSE at both doses (0.53 and 5.3 mg/kg) was found to be effective in reducing IgE titre in both blood serum and BAL fluid of treated animals. Decreased concentration of IgE can be related to suppression of histamine release from mast cells and demonstrating anti-allergic role of the plant. *C. serratum* contains a pentacyclic triterpenoid saponin, icosahydropicenic acid, that shows significant antihistaminic activity, antiallergic activity, and mast cells stabilization properties in in vitro studies [40].

Inflammatory cells in asthmatic individuals are capable of producing several folds more oxygen free radicals and nitrites by autooxidation of nitric oxide molecule (NO) than normal people [41]. Increase in generation of NO, called ‘nitrosative stress’, contributes to ongoing process of inflammatory process in airways. The nitric oxide is produced during production of the L-citrulline from L-arginine by *i*NOS (inducible nitric oxide synthase) that, in turn, inhibits the arginase activity. *i*NOS is also induced by biochemicals, such as TNF-α, IL-1ß and stimulates formation of nitric oxide. Nitrate (NO_3_−) and nitrite (NO_2_−) radicals are metabolite products of NO oxidation [42]. Studies report that acidic pH in asthmatic airways may facilitate conversion of nitrite to NO. NO_2_^−^/ NO_3_^−^ and FENO levels may be considered as biological markers to relate it with intensity of allergic sensitization [43]. In the current study, investigating the role of CSE on NO and nitrites in blood and BAL fluid of allergen sensitized rats, the crude drug extract was found to reduce levels of both gaseous radicals in significant manner.

Potential role of *C. serratum* ethanolic (70%) extract observed in our study can be related to presence of phenolic and steroidal compounds present in the plant mentioned elsewhere. We had chosen 70% ethanol for extraction and biological studies on the basis of our preliminary studies that show maximum % extractive yield, obtained with ethanol: water (7: 3) mixture. In addition, maximum flavonoid and phenolic content (w/w) was also analyzed in CSE. Nevertheless, due to lack of analytic facilities in our lab, it was not possible to quantitatively standardize the crude herbal extract for specific biomarkers. However, collective piece of evidence shows that pentacyclic tripterpenoids, oleanolic acid, ursolic acid, queretaroic acid exhibit large physiological effects and antiasthmatic and antiinflammatory activities in laboratory animals. In preclinical studies, oleanolic acid was able to reduce airway resistance, eosinophil infiltration, interleukin-5, 13, 17 and ovalbumin-specific IgE levels by upregulating of expression of T-bet and Foxp3. The phenolic compound intervenes in cell transduction pathways mediated via GATA-3 and RORγt mechanism, major pathways in maintaining balance of T_H_1/T_H_2 in allergic conditions [44]. Another phenolic, ursolic acid is markedly active in decreasing oxidant stress and cell apoptosis in airway and smoke-induced rat model of emphysema [45].

A flavonoid largely present in *C. serratum* is scutellarein. This compound is capable of inhibiting IL-4, TNF-α, and RNA expression of NO synthase (*i*NOS) and other signaling transducer proteins such as p85/PI3K, IκBα, Src, AKT [46]. Similarly, apigenin has the ability to modulate levels of proinflammatory mediators IL-1ß, TNF-α, NO, lymphocytes, and neutrophil levels in in vitro studies [47]. Accumulating all the evidence, we can presume that presence of phenolic bioactives in *C. serratum* could be collectively responsible for therapeutic role of herbal drug in bronchial asthma. Similar pharmacological effects in animal models of asthma have been observed with phytosterols, stigmasterol, and β-sitosterol [48, 49]

**Figure 5:**
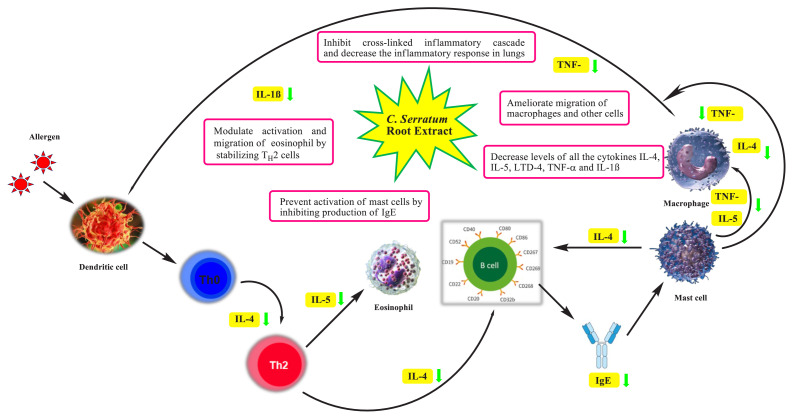
Proposed mode of action of *C. serratum* extract.

Our findings suggest that CSE could exert antiasthmatic effects in experimental rats by regulating T_H_2 related cytokines production in lungs, mainly IL-4, IL-5, and others. Suggested mode of action of *C. serratum* is depicted in Figure 5. Reduced levels of T_H_2 derived cytokines could inhibit secretion of IgE and thereby suppress degranulation of mast cells and release of several cytokines, chemokines that might ameliorate airway inflammation cascade and ROS generation in lungs [50]. Chemokines act as chemoattractant for eosinophils and other inflammatory cells in rat airways. These studies demonstrate the multiple targets underlying the pharmacological effect of our herbal drug in bronchial asthma. Despite promising results, the clinical approach towards use of *C. serratum* in asthma therapy needs detailed pharmacological studies at cellular and molecular levels.

## 4. Conclusion

The results of our study statistically proves and suggests the potential role of *C. serratum* root extract in treatment and management of allergic asthma by attenuating ongoing inflammatory processes mediated through inhibiting the release of inflammatory and proinflammatory mediators and subsequent infiltration of eosinophils, lymphocytes, neutrophils into lungs airways. Further studies on bioactives present in *C. serratum* may be advised to investigate its mechanism of action at molecular and cellular levels.

## Figures and Tables

**Table 1:** Estimation of total leucocyte count (TLC) and differential cell count in blood.

Groups	N	S	S+D	S+CSE 1	S+CSE 2
Total cells	9.33 ± 0.42	17.38 ± 1.11###	11.54 ± 0.41***	14.02 ± 0.11*	16.87 ± 0.54*
Eosinophils	0.66 ± 0.04	4.84 ± 0.34###	1.95 ± 0.25**	3.62 ± 0.51*	4.54 ± 0.82*
Lymphocytes	10.82 ± 0.39	7.25 ± 0.75^##^	10.58 ± 0.34*	8.90 ± 0.11 ^ns^	11.11 ± 1.67*
Macrophages	6.43 ± 0.58	6.39 ± 0.56^NS^	6.38 ± 0.33^ns^	6.33 ± 0.52^ns^	6.55 ± 1.45^ns^
Neutrophils	0.36 ± 0.07	4.21 ± 0.44###	1.97 ± 0.25**	3.67 ± 0.51^ns^	4.96 ± 0.38*

Effect of CSE on inflammatory migratory cells in blood of rats after various treatments. Values represent mean ± SEM (n = 6);

* p < 0.05,

** p < 0.01,

*** p < 0.001 and ns, compared to the asthma control (S) group;

### p < 0.001 and NS compared to normal control (N) group.

**Table 2:** Estimation of total leucocyte count (TLC) and differential cell count in BAL fluid (×10^5^ cells/mL).

Groups	N	S	S + D	S + CSE 1	S + CSE 2
Total cells	7.07 ± 0.49	11.23 ± 0.85###	4.86 ± 0.45***	9.02 ± 0.66*	7.83 ± 0.48**
Eosinophils	0.53 ± 0.52	8.47 ± 0.42###	3.09 ± 0.43***	6.83 ± 0.16*	5.15 ± 0.43*
Lymphocytes	9.89 ± 0.46	18.41 ± 0.75###	10.19 ± 0.69***	14.33 ± 0.11*	12.32 ± 0.28*
Macrophages	5.32 ± 0.49	12.22 ± 0.76###	6.59 ± 0.79***	10.45 ± 0.51*	8.76 ± 0.19*
Neutrophils	0.57 ± 0.04	5.67 ± 0.44###	1.19 ± 0.07***	4.72 ± 0.51 ^ns^	3.01 ± 0.39*

Effect of CSE on inflammatory migratory cells in BAL fluid of rats after various treatments. Values represent mean ± SEM (*n = 6*);

* p < 0.05,

** p < 0.01,

*** p < 0.001 and ns compared to the asthmatic control (S) group;

### p < 0.001 and NS compared to normal control (N) group.
